# Removal of Glyphosate from Water by Adsorption Using the Zeolitic Imidazolate Framework ZIF-8: Characterization of Equilibrium Isotherms and Adsorption Kinetics

**DOI:** 10.1021/acsomega.5c13163

**Published:** 2026-03-18

**Authors:** Crivian Pelisser, Gustavo Lopes Colpani, Vinícius T. Orso, Débora C. Leite, Jaqueline Scapinello, Charles-Francois de Lannoy, Daniel Eiras

**Affiliations:** † Graduate Program in Chemical Engineering, 28122 Federal University of Paraná, Chemical Engineering Department - Rua Coronel Francisco H. dos Santos, s/n° - 81530-000 Curitiba, Parana, Brazil; ‡ SENAI Santa Catarina University Center, UniSENAI − Rua Frei Bruno 201E - 89803-785 Chapecó, Santa Catarina, Brazil; § Community University of Chapecó Region - Unochapecó, Graduate Program in Environmental Science, Servidão Anjo da Guarda, 295 D - Chapecó 89809-900, Santa Catarina, Brazil; ∥ Community University of Chapecó Region - Unochapecó, Graduate Program in Technology and Innovation Management, Servidão Anjo da Guarda, 295 D - Chapecó 89809-900, Santa Catarina, Brazil; ⊥ Department of Food Engineering and Chemical Engineering, State University of Santa Catarina - UDESC, BR 282, km 573, Pinhalzinho 89870-000, Santa Catarina, Brazil; # Department of Chemical Engineering - Main Street West, 3710 McMaster University, 1280 - L8S 4L7 Hamilton, Ontario, Canada

## Abstract

Glyphosate, an anionic organophosphorus herbicide, is frequently detected in surface waters alongside other emerging contaminants (ECs). Effective removal strategies are essential to reduce their environmental impact. This study synthesized a metal organic framework (MOF) called Zeolitic Imidazolate Framework-8 (ZIF-8), a material recently recognized for its exceptional versatility, and employed it for the removal of glyphosate from aqueous systems via adsorption. ZIF-8 particles were synthesized using zinc nitrate hexahydrate as the metal source, 2-methylimidazole as the ligand, and methanol as the solvent. ZIF-8 was characterized by XRD, BET, zeta potential, and FTIR. Adsorption data were well described by both Langmuir and Freundlich models (*R* ^2^ = 0.99), indicating contributions from monolayer and multilayer adsorption mechanisms. The maximum adsorption capacity was 67.67 mg/g at 308 K according to the Langmuir model. The adsorption mechanism involved a combination of physisorption and chemisorption and was influenced by coexisting chloride anions. Thermodynamic analysis indicated that glyphosate adsorption is endothermic and involves chemical interactions between glyphosate and ZIF-8. FTIR spectra showed new bands corresponding to the phosphoryl group after adsorption, confirming glyphosate binding. Overall, this study demonstrates that ZIF-8 is a promising adsorbent for the removal of ECs, offering an effective strategy for environmental protection.

## Graphical abstract

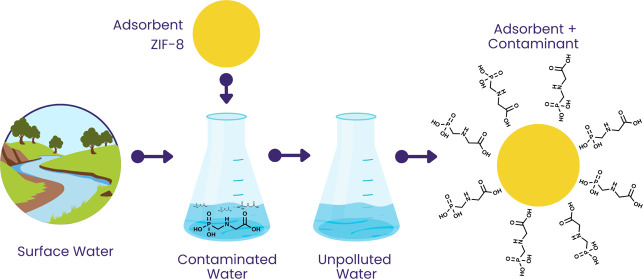

## 1 Introduction

Anthropogenic activities are introducing numerous contaminants into surface and groundwater, compromising water quality and posing risks to public health.^1^ These contaminants, often referred to as Emerging Contaminants (ECs), include pesticides, pharmaceutical products, microplastics, and other substances typically found at low concentrations (ng/L to μg/L) in water and wastewater,^2,3^ which are difficult to remove by conventional treatment methods.^4–6^ Globally, almost 3 billion kilograms of pesticides are applied each year, presenting a significant long-term risk due to their persistence and potential for accumulation in the environment.^6,7^ Although pesticides enhance agricultural productivity and food quality,^5,8,9^ exposure has been linked to increased risk of several diseases.^10–16^ Consequently, developing effective strategies to remove ECs and ensure safe drinking water is critical.

Among pesticides, glyphosate (*N*-phosphonomethyl-glycine), first reported in 1971 by John E. Franz, is widely used due to its high efficiency, water solubility, and broad-spectrum control.^17^ China is the world’s largest producer and exporter of glyphosate technical material, with production exceeding 500,000 t, primarily supplying countries such as the United States, Brazil, and Argentina while domestic consumption reached approximately 128,752 t in 2024.^18^ In the United States, glyphosate remains the dominant herbicide due to its broad-spectrum efficacy.^19,20^ Brazil shows similarly intensive use, where glyphosate represented about 41% of total pesticide consumption in 2024 (231,888 t).^21^ In contrast, the European Union applies a more restrictive regulatory framework; glyphosate has been authorized since 2002 and was most recently reapproved to permit its use until December 2033 under specific risk-mitigation measures.^22^

Glyphosate can degrade microbially to aminomethylphosphonic acid (AMPA), which may persist for up to 1000 days, and both compounds have been detected in soils, sediments, surface water, groundwater, and food.^23,24^ Several methods have been explored for glyphosate removal or degradation, including hydrolysis, advanced oxidation processes, nonthermal plasma, photochemical degradation, adsorption, and membrane separation.^25–29^ Additionally, glyphosate is particularly challenging to remove because of its small molecular size (≈10 Å).^25^

Adsorption is a highly effective strategy for removing emerging contaminants (ECs),^30–33^ operating under ambient pressure, minimizing the generation of concentrated aqueous waste streams, and allowing for material regeneration and reuse.^34^ Various adsorptive media have been applied for the removal of ECs at trace concentrations. Among these, granular activated carbon (GAC) has been widely studied and shown to be effective for long-chain compounds (>C8), although its efficiency decreases for short-chain species (<C6) such as glyphosate.^35,36^ Ion-exchange resins represent an accessible option, although their service life may be limited.^34^ Alternatively, metal-organic frameworks (MOFs) have attracted considerable attention due to their remarkable properties for adsorption applications. Among them, the zeolitic imidazolate framework, ZIF-8, is noteworthy, as it has been widely applied for the removal of ECs owing to its high surface area and tunable pore size.^30,37,38^ Previous studies have evaluated various adsorbents, including activated carbon (AC), AC modified with silver nanoparticles (AC@AgNPs),^39^ goethite nanoparticles,^40^ resin D301 ^41^, and MOFs such as MIL-101.^42^ Functionalization of MIL-101 with amino groups (NH_2_-MIL-101) significantly enhanced glyphosate adsorption (431 mg/g compared to 296 mg/g for unmodified MIL-101 at pH 4), with adsorption equilibrium best described by the Langmuir model and kinetics following a pseudo second-order model.^42^ Another application of ZIF-8 is its ability to act as a host for larger organic molecules. ZIF-8 has shown good performance in caffeine encapsulation, achieving a loading of 28 wt % and a controlled release over approximately 27 days, attributed to interaction forces such as van der Waals forces and hydrogen bonding.^43^ Similarly, ZIF-8 has been used as a pH-responsive carrier for metformin, allowing substantial drug loading and accelerated release under acidic conditions.^44^

A ZIF-8 composite (Fe@ZIF-8) was employed for glyphosate sensing, showing the formation of a stable coordination complex between the Fe^3+^ sites in Fe@ZIF-8 and the phosphonic acid and carboxyl functional groups of the glyphosate molecule. A strong linear relationship was observed between the fluorescence difference (Δ*F*) and the natural logarithm of the glyphosate concentration over the range of 0.05–3 mg/L (*R* ^2^ = 0.99), with a detection limit of 0.022 mg/L, indicating high sensitivity for glyphosate detection.^45^

Research on glyphosate adsorption using ZIF-8 remains scarce, with only one study reported to date. This study investigated glyphosate removal using ZIF-8 and Fe_3_O_4_@ZIF-8 with an average particle size of approximately 1 μm and a surface area of 427 m^2^/g. The particles were produced by using a 24 h synthesis procedure involving multiple drying and washing steps followed by resuspension. Glyphosate concentrations in the range of 0–120 ppm were tested, and quantification relied on colorimetric methods.^46^ In the present work, ZIF-8 nanoparticles (50 nm) with a high surface area (1620 m^2^/g) were applied to adsorb glyphosate from water. The synthesis methodology in this study is fast (1 h) and easy to reproduce. It is also known that the properties of ZIF-8 can be influenced by various synthesis parameters, including the molar ratio between the metal and ligand, type of solvent, reaction temperature, mixing, and reaction time.^47,48^ Therefore, the ZIF-8 synthesized in this study exhibits properties different from those reported in previous studies. Our study applied high-performance liquid chromatography coupled with mass spectrometry (LC–MS/MS) to provide a more sensitive and reliable assessment of adsorption performance. Additional investigations are necessary, particularly because ZIF-8 properties are influenced by the synthesis methodology, which can affect adsorption performance. ZIF-8 has shown potential in contaminant removal; its interaction with glyphosate, adsorption kinetics, and thermodynamic behavior remains insufficiently explored. This study aims to address these gaps by evaluating the adsorption efficiency of ZIF-8 for glyphosate removal, its thermodynamic behavior, and the dominant adsorption mechanism.

In this context, the feasibility of using ZIF-8 for the adsorption of glyphosate from an aqueous medium was evaluated. ZIF-8 was synthesized through a rapid room-temperature process, which is the most promising technique for large-scale production. The structure of ZIF-8 was initially characterized by using XRD, SEM, BET, FTIR, DLS, and zeta potential analysis. Following this, adsorption tests were conducted, and the effects of the pH, temperature, ionic strength, and adsorbent dosage were investigated. Thermodynamic studies were performed, and a mechanism for glyphosate adsorption onto ZIF-8 was proposed. The results show that ZIF-8 is a viable candidate for glyphosate removal from water and that the adsorption is governed by chemical interactions that can be electrostatic and nonelectrostatic, with evidence of chelation between glyphosate and zinc moieties within ZIF-8.

## 2 Materials and Methods

### 2.1 Chemicals

Zinc nitrate hexahydrate and 2-methylimidazole were purchased from Sigma-Aldrich, Germany. Methanol was purchased from Quimica Moderna, Brazil, and technical glyphosate 95% (Gly) was used as received. Analytical grade glyphosate (Gly) (Analytical Standard) was acquired from Sigma-Aldrich (St. Louis, USA). Formic acid (98–100% w/w) was obtained from LiChropur (Darmstadt, Germany); LC-grade acetonitrile was supplied by LiChrosolv (Darmstadt, Germany), and deionized water was purified using a Milli-Q SQ200 system (Millipore, Darmstadt, Germany). The LC passivation solution containing 1760 μg/mL medronic acid was acquired from Restek Corp. (Bellefonte, PA, USA).

### 2.2 Synthesis of ZIF-8 Nanoparticles

ZIF-8 was synthesized by dissolving 80 mmol of 2-methylimidazole in 60 mL of methanol and 10 mmol of zinc nitrate hexahydrate in 120 mL of methanol, following the procedure initially established by Cravillon et al.^49^ The two solutions were stirred until a complete dissolution of the reagents was achieved. The zinc solution was then introduced into the imidazole solution and thoroughly mixed. The reaction mixture was maintained for 60 min under magnetic stirring at 1000 rpm. Subsequently, the product was repeatedly washed and centrifuged with methanol. The samples were then dried in an oven at 60 °C for 3 h.

### 2.3 Characterization of ZIF-8 Nanoparticles

XRD analysis (Bruker diffractometer, D8 Advance) was employed to characterize the crystalline structure. The size and morphology of the particles were determined by Scanning Electron Microscopy (SEM) analysis (JEOL - JEM 1200EX-II). The elemental composition of the ZIF-8 nanoparticles was examined by energy-dispersive spectroscopy (EDX) using an EDAX, model Elect Plus. The BET isotherms analysis (Quantachrome - Autosorb iQ) was conducted to determine the specific surface area of the synthesized ZIF-8, as well as the pore volume and pore size. Dynamic light scattering (DLS) (Zetasizer Nano ZS) analysis was employed to determine the size and behavior of ZIF-8 in water. The suspension was progressively diluted until the measurements stabilized, ensuring that the smallest particles in the suspension were accurately characterized. Zeta potential (ZP) was used to measure the surface charge of ZIF-8 and glyphosate under different pHs.

### 2.4 Evaluation of Adsorption Performance

For the equilibrium test, working solutions with concentrations of 2, 4, 6, 8, 10, 15, 20, 25, 30, and 50 mg/L of Gly were prepared in deionized water. Subsequently, 20 mg of ZIF-8 was added to 50 mL of each Gly solution. The suspensions were left under agitation (200 rpm) for 120 min at 25 °C. Next, the suspensions were filtered by using a 0.2 μm polytetrafluoroethylene (PTFE) syringe filter to remove the adsorbent.

The adsorption capacity of the adsorbent (*q*_e_) and removal efficiency (*R*) were determined by eqs 1 and 2

1 $$q_{e}=\frac{\left(C_{0}-C_{e}\right)V}{m}$$

2 $$R=\frac{\left(C_{0}-C_{e}\right)}{C_{0}}\times 100$$

where *q*_e_ (mg/g) represents the adsorption capacity, *C*_0_ represents the initial concentration of Gly (mg/L), *C*_e_ (mg/L) is the concentration of the Gly at equilibrium, *V* (*L*) is the volume of the solution, *m* (*g*) represents the mass of the ZIF-8 applied, and *R* (%) represents the removal efficiency of Gly. The isotherm experiments at different temperatures were conducted in triplicate, while the kinetic, mass of ZIF-8, and salt influence experiments were performed in duplicate. The average values plus the standard deviations were reported.

The adsorption isotherm describes the relationship between adsorption and concentration at a constant temperature.^50^ Among the commonly used models are the Langmuir isotherm,^51^ which describes monolayer adsorption, and the Freundlich isotherm,^52^ which considers rough and heterogeneous surfaces with multilayer adsorption. The corresponding equations are provided in eqs 3 and 4

3 $$Langmuir:q_{e}=\frac{q_{\max}k_{L}C_{e}}{1+k_{L}C_{e}}$$

4 $$Freundlich:q_{e}=k_{F}+C_{e}^{1/n}$$

where *q*_e_ (mg/g) represents the amount of solute adsorbed per gram of adsorbent at equilibrium, *q*_max_ (mg/g) is the maximum adsorption capacity, *k*_L_ (L/mg) is the Langmuir constant associated with the binding energy in the adsorption process; n is a constant related to surface heterogeneity, and *k*_F_ (mg/g­(mg/L)^−1/*n*^) is the Freundlich adsorption capacity constant.

Furthermore, the Langmuir dimensionless separation factor (*R*_L_) expressed with eq 5 was calculated to understand whether the Gly adsorption process was a favorable or unfavorable mechanism.

5 $$R_{L}=\frac{1}{1+k_{L}C_{0}}$$

when the *R*_L_ value is between 0 and 1, the adsorption process is favorable and the isotherm exhibits a concave shape. However, if the value is greater than 1, adsorption is unfavorable.^53^

The adsorption process involves the attraction between the adsorbent and the adsorbate, allowing for the occurrence of physical attraction, chemical attraction, and internal and external diffusion mechanisms.^50^ For the adsorption kinetics experiment, the initial Gly concentration was maintained at approximately 30 mg/L, with a volume of 50 mL. Measurements were performed at time intervals of 1.0, 3.0, 5.0, 10.0, 20.0, 30.0, 50.0, 80.0, and 120.0 min. To clarify the adsorption mechanism and determine the process’s controlling steps, the experimental data were analyzed using kinetic models, specifically the pseudo-first-order, pseudo-second-order, and intraparticle diffusion models, as summarized in Table 1.^54^ where *q*_e_ and *q*_t_ are the quantities adsorbed per gram of adsorbent at equilibrium and at time *t*, respectively (mg/g); *k*_1_ is the rate constant of the pseudo-first-order adsorption (1/min); *k*_2_ is the pseudo-second-order adsorption constant (g/(mg.min); *k*_d_ is the intraparticle diffusion coefficient (mg/g.min^–0,5^); *C* is the constant related to diffusion resistance (mg/g). The parameter *C* allows for the estimation of the boundary layer’s thickness, as it is directly proportional to its value. When *C* = 0, the adsorption process is governed by intraparticle diffusion, indicating a minimal influence from other interactions at the boundary layer. Conversely, when *C* ≠ 0, the adsorption process involves more complex dynamics.^54^

**Table 1:** Kinetic Models Commonly Used to Explain the Adsorption Process

kinetic model	equation	limiting stage	reference
pseudo-first-order	$q_{t}=q_{e}\left(1-e^{1-k1t}\right)$ 6	physisorption	^54,55^
pseudo-second-order	$\frac{t}{q_{t}}=\frac{1}{{k_{2}q}_{e}^{2}}+\frac{t}{q_{e}}$ 7	chemisorption	^54^
intraparticle diffusion	8 $q_{t}=k_{d}t^{0.5}+C$	intraparticle diffusion	^56^

### 2.5 Influence of Temperature, Salt Concentration, and Adsorbent Dosage

ZIF-8 was analyzed a second time considering temperatures of 25, 35, and 45 °C using 20 mg of ZIF-8 in 50 mL of Gly solution at 30 mg/L. Moreover, to calculate the thermodynamic parameters (the apparent enthalpy change, entropy change of adsorption, and free energy change, Δ*H*°, Δ*S*°, and Δ*G*°, respectively), eqs 9 and 10 were applied5 ^50^

9 $$\ln\left(K_{c}\right)=\frac{\Delta S^{{}^{\circ}}}{R}-\frac{\Delta H^{{}^{\circ}}}{RT}$$

10 $${\Delta G}^{{}^{\circ}}=\Delta H^{{}^{\circ}}-T\Delta S^{{}^{\circ}}$$

where *R* is the universal gas constant (8.314 J/(mol K)), *T* is the temperature (K), and *K*_c_ is the thermodynamic equilibrium constant. The values of Δ*H*° and Δ*S*° were calculated from the slope and *y*-intercept from the plot of ln (*K*_c_) vs 1/*T*.

The influence of salt presence and adsorbent dosage was investigated using 50 mL of an approximately 30 mg/L glyphosate solution at 25 °C, 200 rpm, and 120 min of adsorption. NaCl was added to a glyphosate solution at molar concentrations of 0.3 and 0.5 M to analyze effect of salt and ionic strength on adsorption capacity. Similarly, the effect of adsorbent dosage on adsorption capacity and Gly removal efficiency was examined by adding varying doses of ZIF-8 between 20 and 50 mg.

### 2.6 Quantification of Glyphosate

A stock standard solution of Gly (with concentrations of 1000 μg/L) was prepared in water/acetonitrile (90:10, v/v) and stored at 4–8 °C in polypropylene volumetric flasks. Calibration curve standard solutions (5, 10, 25, 50, 100, 250, and 500 μg/L) for quantitation were prepared from the working stock solution in water/acetonitrile (90:10, v/v). The standard solutions were filtrated through syringe Durapore PVDF membranes with pore diameters of 0.22 μm from Millipore and transferred to a 1.5 mL vial.

Before the injection, the samples were filtrated through syringe Durapore PVDF membranes with pore diameter of 0.22 μm from Millipore, diluted 100-fold with water/acetonitrile (90:10, v/v), and transferred to a 1.5 mL vial prior to injection into the LC–MS/MS system. The instrumental analysis was performed by using a Shimadzu system (LC-40D XR), with a CTO-40C column oven, coupled with an LCMS-8050 triple quadrupole mass spectrometer (Kyoto, Japan). The software LabSolutions (version 5.128) was used for the control and data acquisition. Chromatographic separation was obtained by using a Raptor Polar X (2.7 μm, 30 × 2.1 mm) column from Restek (Bellefonte, PA, USA). The mobile phases consisted of 0.5% (v/v) formic acid in water (A) and 0.5% (v/v) formic acid in acetonitrile (B). The column temperature was maintained at 35 °C, 10 μL of sample injection, and a gradient elution was performed at 0.6 mL/min as follows: 0.0–1.5 min 60% B; 1.5–2.5 min from 60% to 5% B; 2.5–6.5 min 5% B; and 6.5–10.0 min 60% B. Retention time was approximately 5 min for glyphosate.

To prevent the chelation or interaction of glyphosate with metal ions, a passivation procedure was performed on the LC system before analysis. A total of 10 full-loop injections of 50 μL of the medronic acid solution were performed without a column, and 5 full-loop injections of 2 μL of passivation solution with the column were performed using the mobile phases of the analytical method.

The mass spectrometer determination was performed with negative electrospray ionization operated in the multiple reaction monitoring mode using the following *m*/*z* transitions: 168.2 → 63.0 (quantifier ion, CE = 23 V) and 168.2 → 81.0 (confirmation ion, CE = 15 V). The selected parameters of the ionization source were as follows: the interface voltage was 4.0 kV, the nebulizing gas flow was 3.0 L/min, the drying and heating gas flow was 10 L/min, the interface temperature was 300 °C, the desolvation temperature was 526 °C, the DL temperature was 250 °C, the heat block temperature was 400 °C, and the CID gas pressure was 270 kPa.

## 3 Results and Discussion

### 3.1 Characterization of ZIF-8 Nanoparticles

The XRD spectra of synthesized ZIF-8 nanoparticles exhibited peaks at 2θ angles of 7.3, 10.4, 12.7, 14.7, 16.4, 18.0, 24.5, and 26.6° (Figure 1a).^49,57,58^ SEM images of ZIF-8 (Figure 1b) showed rhombic particles, consistent with the results reported by Cravillon et al.^49^ The average particle size of the synthesized particles was 50 nm. Different particle sizes have been reported in the literature, and this difference can be attributed to the synthesis methodology. The particle size of ZIF-8 can be controlled by adjusting various synthesis parameters, as the molar ratio between the metal and ligand, solvent type, reaction temperature, and reaction time.^47,48^ The chemical composition was evaluated by using EDS on a crystal of ZIF-8 (Figure 1c), where carbon was the predominant element (47%), and zinc and nitrogen were present in similar amounts (23%),^59,60^ consistent with ZIF-8 materials.^59,60^ The ZIF-8 surface area, pore volume, and pore diameter were found to be 1620 m^2^/g, 0.632 cm^3^/g, and 1.3 nm, respectively (Figure S1 and Table S1), which are in agreement with values reported in the literature.^61–63^ This result confirms that the structure obtained is characteristic of ZIF-8 material, as previously identified by XRD, SEM, and EDS.

**Figure 1:**
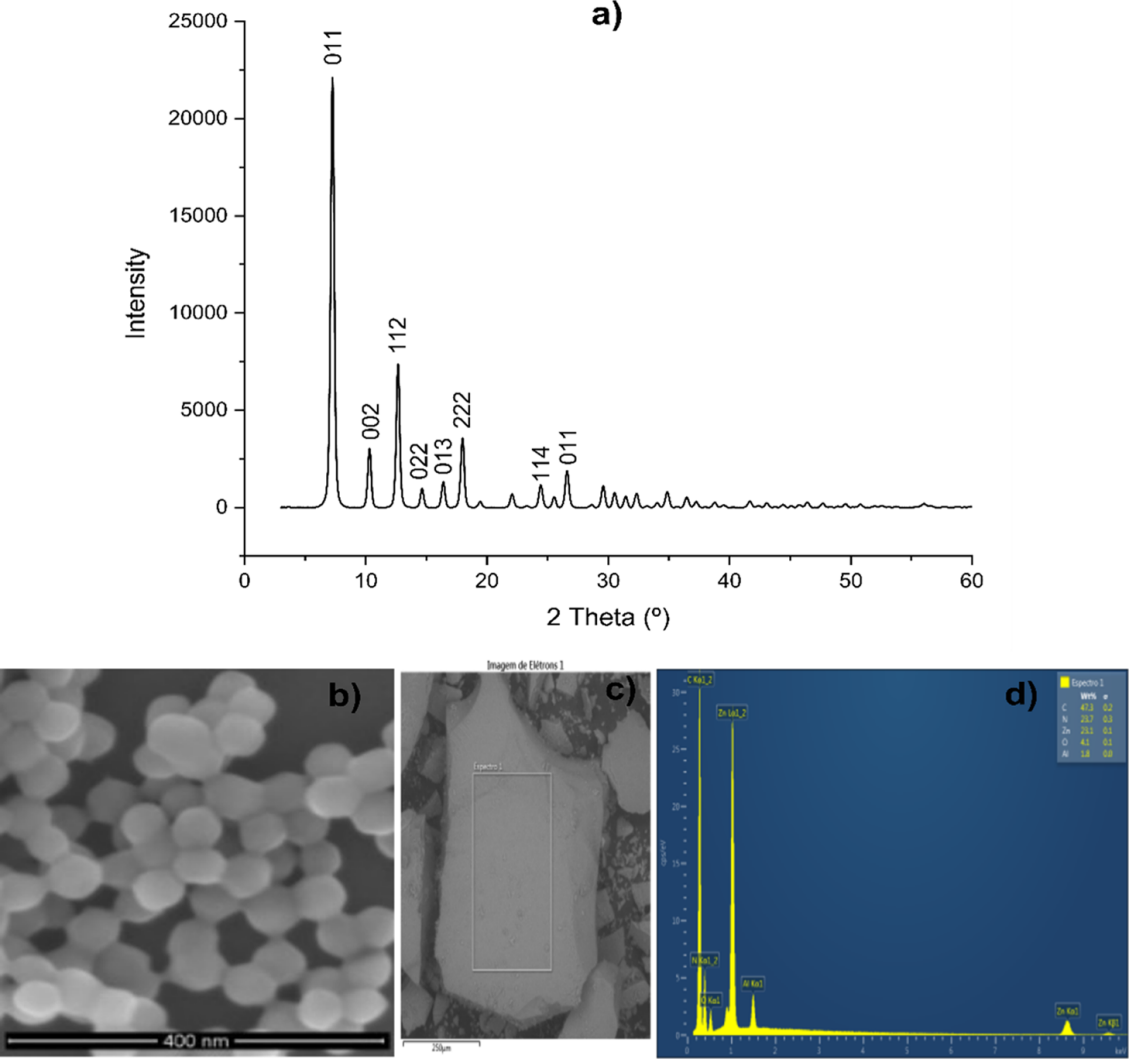
(a) Diffractogram of ZIF-8; (b) micrographs of ZIF-8 with magnification of 175,000 times; (c) ZIF-8 crystal with the selected area indicated by the rectangle; (d) chemical distribution of ZIF-8 in the area indicated by the rectangle.

The water stability of ZIF-8 was evaluated after 24 h of exposure to water (Figure S2 and Table S2). Following the water treatment, an additional diffraction reflection appeared at 10.95°. Previous studies have reported that contact with water can induce new reflections in the 10–11° range, which are attributed to slight structural distortions or the formation of hydrated or intermediate phases, while the overall integrity of the parent ZIF-8 framework remains largely preserved.^64^

### 3.2 Adsorption Isotherms

The parameters of the isotherm’s models for Gly adsorption by ZIF-8 at different temperatures are presented in Table 2, and the adsorption isotherms with 2 h equilibration time are presented in Figure 2.

**Table 2:** Isotherm Model Parameters for Adsorption of Gly on ZIF-8^a^

model	parameter	temperature (K)		
		298	308	318
Langmuir	*q*_max_ (mg/g)	42.93 ± 3.05	67.67 ± 3.24	46.72 ± 5.20
	*k*_L_ (L/mg)	1.21 ± 0.67	1.97 ± 0.94	1.62 ± 0.245
	*R*_L_	0.016	0.010	0.012
	*R* ^2^	0.99	0.99	0.98
Freundlich	*k*_F_ (mg/g(mg/L)^–1/*n*^)	28.57 ± 3.0	^b^	36.91 ± 9.10
	N	7.80	^b^	14.81
	*R* ^2^	0.99	^b^	0.97

a Values are reported as mean ± standard deviation obtained from triplicate experiments.

b The Freundlich Model did not fit.

**Figure 2:**
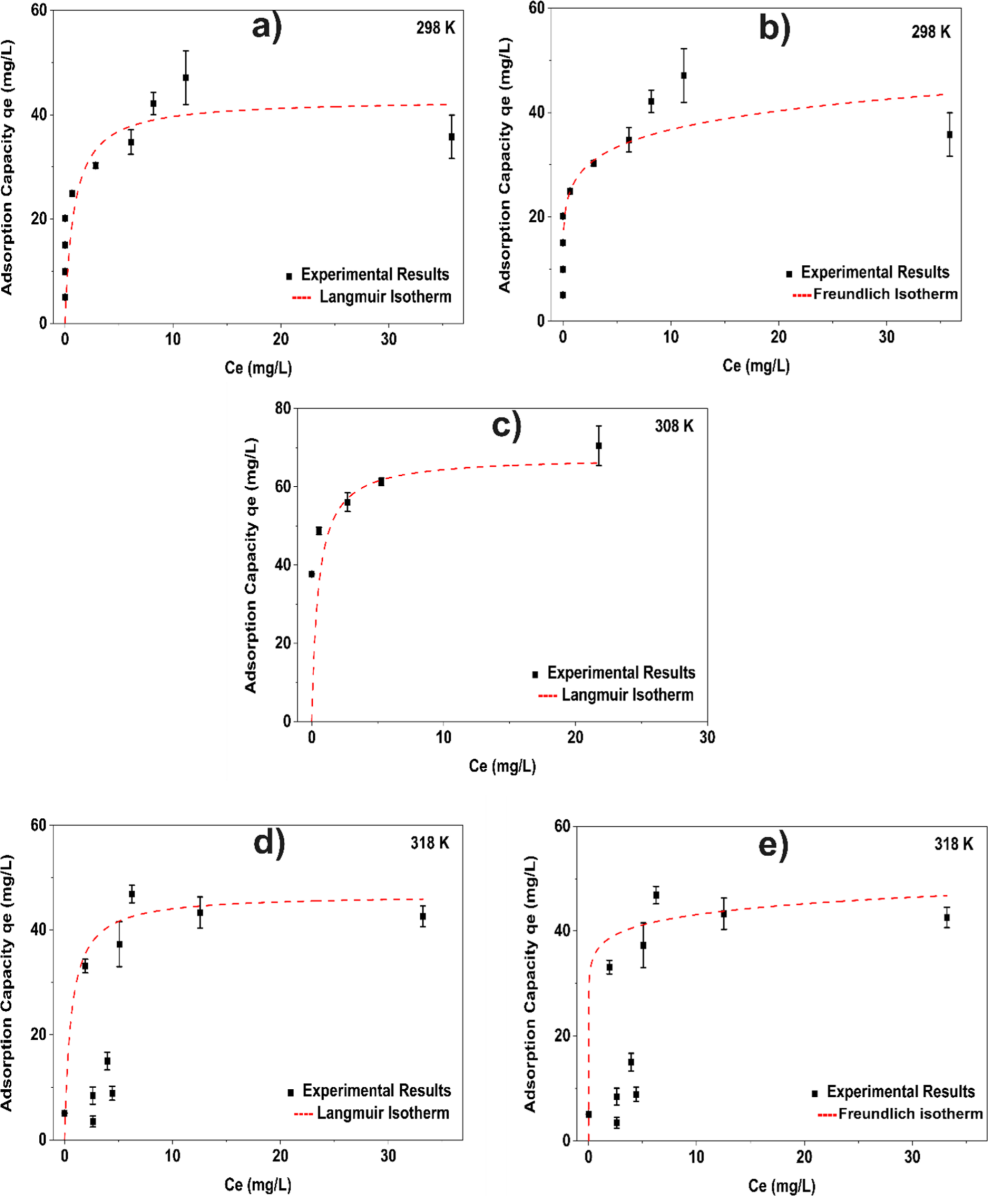
Experimental adsorption capacity of glyphosate onto ZIF-8: experimental data fitted with (a) Langmuir model at 298 K; (b) Freundlich model at 298 K; (c) Langmuir model at 308 K; (d) Langmuir model at 318 K; and (e) Freundlich model at 318 K.

At 298 K, Freundlich and Langmuir models provided an excellent fit to the adsorption data (*R* ^2^ = 0.99), suggesting the coexistence of monolayer and multilayer adsorption mechanisms. With an increase in temperature (308 K), the Langmuir model continued to yield adsorption capacities. At 318 K, the Freundlich and Langmuir models exhibited comparable agreement with the experimental data ((*R* ^2^ = 0.98 and 0.97 respectively), suggesting again the coexistence of monolayer and multilayer adsorption mechanisms. The maximum adsorption capacities derived from the Langmuir model were in close agreement with the experimental observations for all of the temperatures. Moreover, the dimensionless separation factor (*R*_L_) confirmed the favorable nature of the adsorption process, with values ranging between 0 and 1.^53^ It is also important to note that the initial points (2, 4, 6, 8, 10, and 15 mg/L Gly) were not included when applying the Langmuir model, since *C*_e_ was equal to zero; in other words, all the glyphosate was adsorbed up to the concentration of 15 mg/L.

The temperature dependence of adsorption can be further understood through thermodynamic principles. The heat of adsorption decreases with increasing temperature, which can lead to a decrease in adsorption capacity at higher temperatures.^65^ This phenomenon is consistent with the observed decrease in maximum adsorption capacity from 67.67 mg/g at 308 K to 46.72 mg/g at 318 K. The fitting of the adsorption process of Gly with the Langmuir and Freundlich models can be associated with the concept of homotattic behavior, which is often applied to describe adsorption on heterogeneous surfaces. The overall adsorption isotherm is represented as the combination of patch contributions, weighted by their energy distribution and relative proportion. This approach is particularly useful for interpreting ZIF-8 adsorption, as its structural heterogeneity can be approximated through such patch wise homogeneity.^46,66^

The literature reports adsorption isotherms for glyphosate on various materials. Doyle et al.^40^ demonstrated that adsorption on goethite particles could be well described by the Langmuir model, with experiments performed over an 8 h period at pH 5, the *q*_max_ was 277.77 mg/g. Similar results were reported by Ondang et al.,^46^ who studied the adsorption of glyphosate (0–120 mg/L) on ZIF-8 and modified ZIF-8 over a 6 h period, who reported value of 59.91 mg/g for glyphosate adsorption on ZIF-8 at 323 K, which is in agreement with this work.^46^

### 3.3 Kinetic Model

The kinetic parameters for glyphosate adsorption on ZIF-8 were determined using nonlinear fitting of pseudo-first-order, pseudo-second-order, and intraparticle diffusion models. The parameters and correlations presented in Table 3 and the corresponding plots are shown in Figure 3.

**Table 3:** Kinetic Model Parameters for Adsorption of Gly on ZIF-8^a^

nonlinear fit								
pseudo-first-order			pseudo-second-order			intraparticle diffusion		
q_e_ (mg/g)	*k*_1_ (L/min)	*R* ^2^	q_e_ (mg/g)	*k*_2_ (g/(mg.min)	*R* ^2^	*k*_d_	C	*R* ^2^
72.46 ± 7.46	0.01261 ± 0.00161	0.98	114.35 ± 13.64	7.21 × 10^–5^ ± 1.94 × 10^–5^	0.98	3.85 ± 0.19	1 × 10^–14^	0.88

a Values are reported as mean ± standard deviation obtained from triplicate experiments.

**Figure 3:**
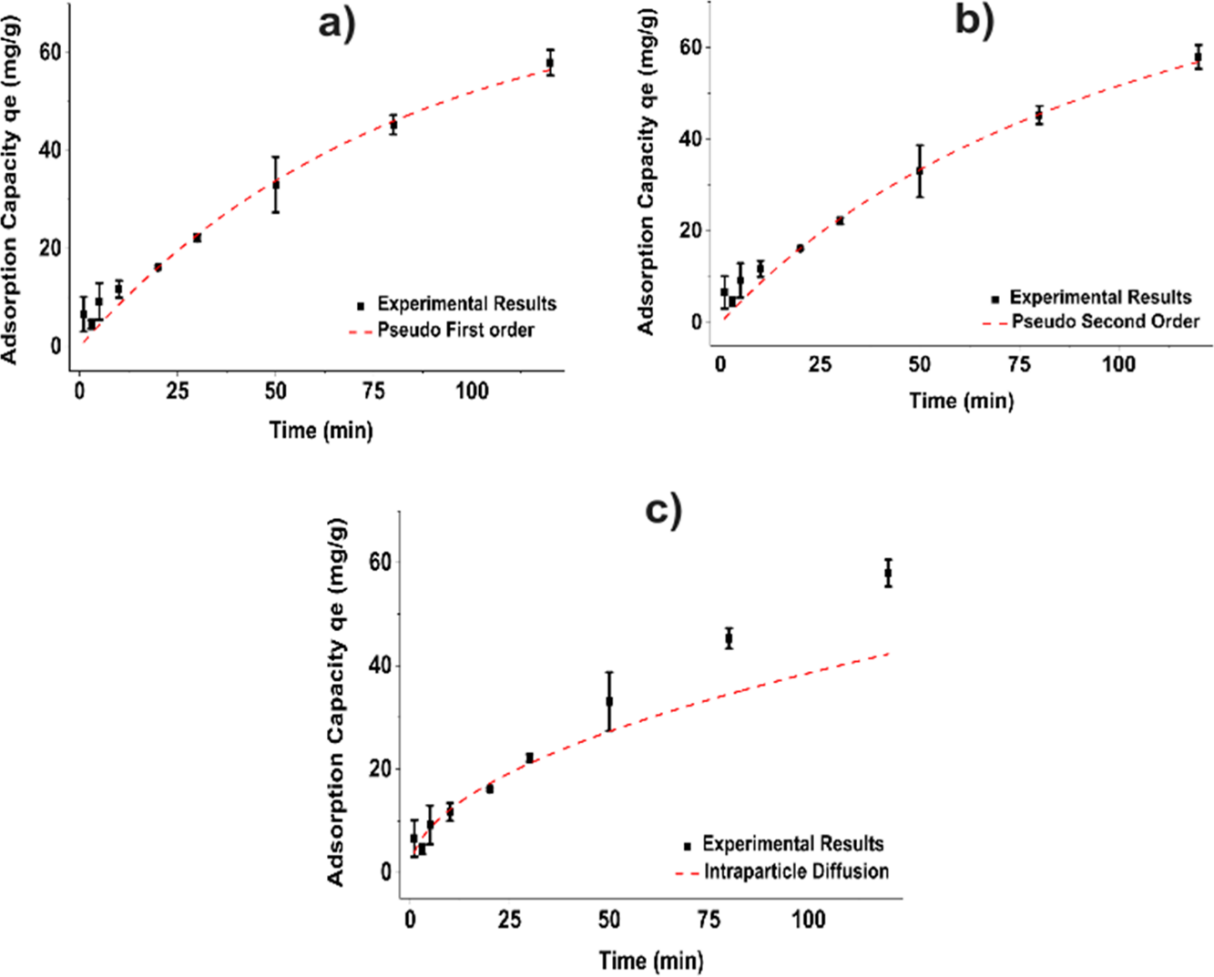
Kinect models for adsorption of Gly on ZIF-8 (a) pseudo-first-order, (b) pseudo-second-order, and (c) intraparticle diffusion.

The models of pseudo-first-order and pseudo-second-order provided excellent fits to the experimental data (*R* ^2^ = 0.98), whereas the intraparticle diffusion model showed a lower correlation (*R* ^2^ = 0.88). The pseudo-first-order model predicted a q_e_ (72.46 ± 7.46 mg/g), which is close to the experimental results at 298 K, suggesting that the adsorption kinetics are partially governed by physisorption processes, typically associated with weak van der Waals interactions. In contrast, the pseudo-second-order model yielded a higher q_e_ value of 114.35 ± 13.64 mg/g, which is interpreted as evidence that chemisorption contributes to the adsorption mechanism. This implies that chemical interactions, such as electron sharing or exchange between glyphosate functional groups and active sites of ZIF-8, may be significant in the adsorption process. The intraparticle diffusion model exhibited a lower *R* ^2^ (0.88), indicating that diffusion is not the primary rate-limiting step. We therefore believe that pseudo-first-order and pseudo-second-order mechanisms are involved in the adsorption process, reflecting contributions from physisorption and chemisorption interactions between glyphosate and the ZIF-8. Several studies have analyzed the adsorption process using ZIF-8 and identified pseudo-second-order kinetics as the most suitable model.^26,67,68^ Nevertheless, this does not necessarily exclude the involvement of a pseudo-first-order adsorption mechanism. In the case of glyphosate adsorption on other materials, the pseudo-second-order model has likewise been reported to provide a better description of the process.^39,40,69^

### 3.4 Effect of Dose and Salinity on Glyphosate Adsorption

Different conditions of adsorbent dose and salinity and detection of glyphosate by FTIR can be seen in Figure 4.

**Figure 4:**
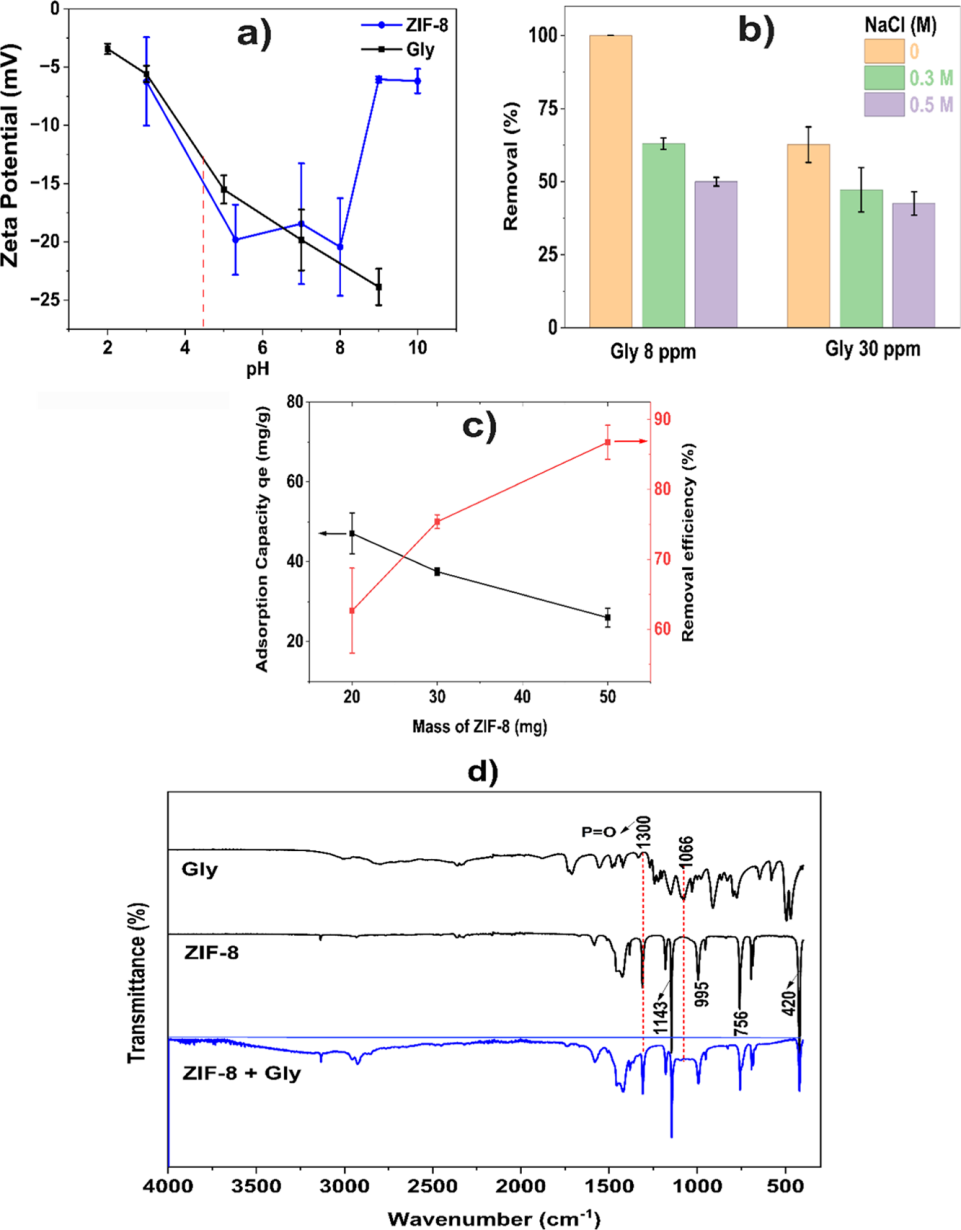
(a) ZP of ZIF-8 and glyphosate at different pH values; (b) effect of NaCl concentration on *q*_e_; (c) adsorbent dose influence on *q*_e_; All in Gly adsorption by ZIF-8; (d) FTIR spectra of glyphosate (pure) and ZIF-8 before and after the adsorption process.

Figure 4a investigates the changes of the ZP of ZIF-8 and glyphosate under different pHs. The ZP of ZIF-8 was negative across all pH levels, with a minimum between pHs 5 and 8. In contrast, the ZP of glyphosate decreased as the pH increased. Glyphosate exhibits zwitterionic behavior, with its dissociation strongly influenced by pH. It contains a carboxyl, an amino, and a phosphonate group, with p*K*_a_ values of approximately 2.6, 5.6, and 10.6, respectively. At strongly acidic pH, glyphosate predominantly exists in its protonated form, whereas increasing the pH results in sequential deprotonation of the carboxyl and phosphonate groups, generating negatively charged species.^54,70^ At pH 8 (this work), glyphosate exists predominantly as negatively charged. The pH was selected because it corresponded to the natural pH established after mixing the solution with the adsorbent. Qiu et al.^71^ found that an acidic environment was more conducive to the adsorption of glyphosate on Fe-WTRs due to the positive charge of the adsorbent and the negative nature of Gly under acidic conditions. The adsorption of glyphosate by ZIF-8 in this pH range is not influenced solely by electrostatic interactions. Since glyphosate is a well-known chelating agent, it is plausible that chelation of glyphosate with zinc acts as an additional adsorption mechanism on ZIF-8.^72,73^ It was also observed by XPS analysis that ZIF-8 is formed by a tetrahedral configuration of imidazole ligands around zinc ions, and the expected Zn/N ratio for this configuration is 0.25 in the bulk of the ZIF-8 particles. This ratio is expected to be greater on the surface due to defects and undercoordinated zinc.^74^

As can be seen in Figure 4b, glyphosate was completely removed at a low initial concentration (8 mg/L), while a decrease in efficiency was observed when the concentration increased (30 mg/L). The presence of NaCl further reduced adsorption efficiency, with more inhibition observed at higher salinity. The decline in removal efficiency in the presence of salt may be attributed to competitive interactions between chloride ions and glyphosate molecules for adsorption sites and electrostatic shielding effects that weaken the adsorbate–adsorbent interaction.^75,76^ In summary, increasing the NaCl concentration negatively affects the adsorption process, as indicated by the gradual decline in removal efficiency, likely due to competitive adsorption between ions.

As shown in Figure 4c, increasing the ZIF-8 dosage enhanced the glyphosate removal efficiency but led to a decrease in adsorption capacity. The improvement in removal efficiency with higher dosages can be attributed to the greater availability of ZIF-8 material, which promotes more extensive adsorption of glyphosate molecules from solution. Conversely, the decline in adsorption capacity arises because the amount of glyphosate adsorbed per unit mass of ZIF-8 decreases as the adsorbate is distributed across an excess of adsorption sites. In addition, particle aggregation at higher dosages may limit the accessibility of active sites, further contributing to the reduction in *q*_e_.^77–79^

The FTIR spectra of ZIF-8 recorded before and after the kinetic test (Figure 4d) show subtle differences that may be associated with the adsorption of glyphosate. After contact with the pesticide, bands that are also found in glyphosate were observed. A broad band around 3467 cm^–1^, which may be related to P–O–H stretching bands in the 1310–1150 cm^–1^ region possibly associated with phosphoryl (P=O) vibrations, and features in the 1200–900 cm^–1^ range that may correspond to P–O stretching.^31^ Additionally, weak bands in the 1650–1550 cm^–1^ region could be related to the carboxylate groups present in the glyphosate structure. However, given the limited sensitivity of FTIR, complementary techniques could be employed to further confirm these results.

### 3.5 Thermodynamic Process

The adsorption process exhibited an Δ*H*° of 45.73 kJ/mol (Table 4), indicating an endothermic process. When Δ*H*° is in the range of 5–10 kJ/mol, the adsorption mechanism is physisorption (van der Waals interactions). When Δ*H*° is in the range of 30–70 kJ/mol, the adsorption is related to chemisorption.^41^ It can be inferred that the adsorption of glyphosate on ZIF-8 occurs mainly through chemical, monolayer, and multilayer adsorption mechanisms, which is consistent with other studies.^40,80^ Thermodynamic analysis indicates that the adsorption is spontaneous at 308 and 318 K (Δ*G*°<0), whereas Δ*G*° values obtained at 298 K is approximately zero, consistent with equilibrium conditions (Δ*G*° ≈ 0) (Figure S3). Δ*S*° positive value indicates irreversible process behavior, which may be correlated to the difficulty of the adsorbate desorption minimizing the potential energy in the system.^46^

**Table 4:** Adsorption Thermodynamics Parameters for *C*_0_ = 6 mg/L

temperature (K)	Δ*G*° (kJ/mol)	Δ*H*° (kJ/mol)	Δ*S*° (kJ/mol.K)	*R* ^2^
298	≈0	45.73	0.15	0.734
308	–1.04			
318	–2.56			

The reusability of adsorbents is an important parameter related to their economic feasibility. Ondang et al.^46^ used ethanol to regenerate ZIF-8 and Fe_3_O_4_@ZIF-8 to release the adsorbed glyphosate. In the second adsorption–desorption cycle, the removal efficiency decreased to approximately 70%, and after three cycles, it further declined to about 25%. The authors attributed this decrease in performance to the incomplete desorption of glyphosate during each regeneration cycle.

ZIF-8 has been demonstrated to be an effective adsorbent for removing glyphosate from water resources, exhibiting a high adsorption capacity of 67.67 mg/g at 308 K. Environmental monitoring studies show that glyphosate concentrations in freshwater systems worldwide vary substantially according to land use and hydrological conditions. In Brazil, reported levels reached up to 2.16 mg/L, particularly in areas influenced by intensive agriculture, while in the United States, concentrations of 427 μg/L were found.^81^ Considering these environmental levels, the adsorption capacity of ZIF-8 indicates that even relatively small amounts of the material could effectively remove glyphosate from contaminated waters, highlighting its potential for mitigating pesticide pollution in diverse aquatic environments.

## 4 Conclusions

The favorable adsorption process, supported by isotherm models and thermodynamic parameters, indicates that ZIF-8 is an efficient and promising material for removing pesticide contamination from water, contributing to sustainable environmental protection. The results show that glyphosate adsorption occurs through a combination of monolayer and multilayer mechanisms, with capacities of 42.93 mg/g (298 K), 67.67 mg/g (308 K), and 46.72 mg/g (318 K) according to the Langmuir model. Kinetic analysis using pseudo-first-order and pseudo-second-order models suggests that both chemical and physical interactions govern the adsorption. Thermodynamic analysis shows that adsorption is spontaneous at 308 and 318 K (Δ*G*° < 0). Future research could explore strategies to further improve the adsorption capacity of ZIF-8 for environmental remediation.

## Supplementary Material



## Notes

N_2_ physisorption data of ZIF-8 (adsorption–desorption isotherms, BET and Rouquerol plots, BET fitting parameters, and pore size distribution calculated by the HK method), XRD patterns before and after 24 h water exposure with corresponding peak fitting results, and additional adsorption data on the effect of temperature on q_e_ and the Van’t Hoff analysis for glyphosate adsorption. (PDF)
