# Supplementary material for: Removal of Glyphosate from Water by Adsorption Using the Zeolitic Imidazolate Framework ZIF-8: Characterization of Equilibrium Isotherms and Adsorption Kinetics
Source: ACS Omega. 2026 Mar 18;11(12):19470–81. doi: 10.1021/acsomega.5c13163 (PMC13044618; doi:10.1021/acsomega.5c13163)
Supplement: Supplementary file 1 [file ao5c13163_si_001.pdf]

Removal of glyphosate from water by adsorption using zeolitic imidazolate framework ZIF-8: characterization of equilibrium isotherms and adsorption kinetics.

Crivian Pelisser[a,b], Gustavo Lopes Colpani[c,d], Vinícius T. Orso[c], Débora C. Leite[c],

Jaqueline Scapinello[e], Charles-Francois de Lannoy[f], Daniel Eiras[a]*

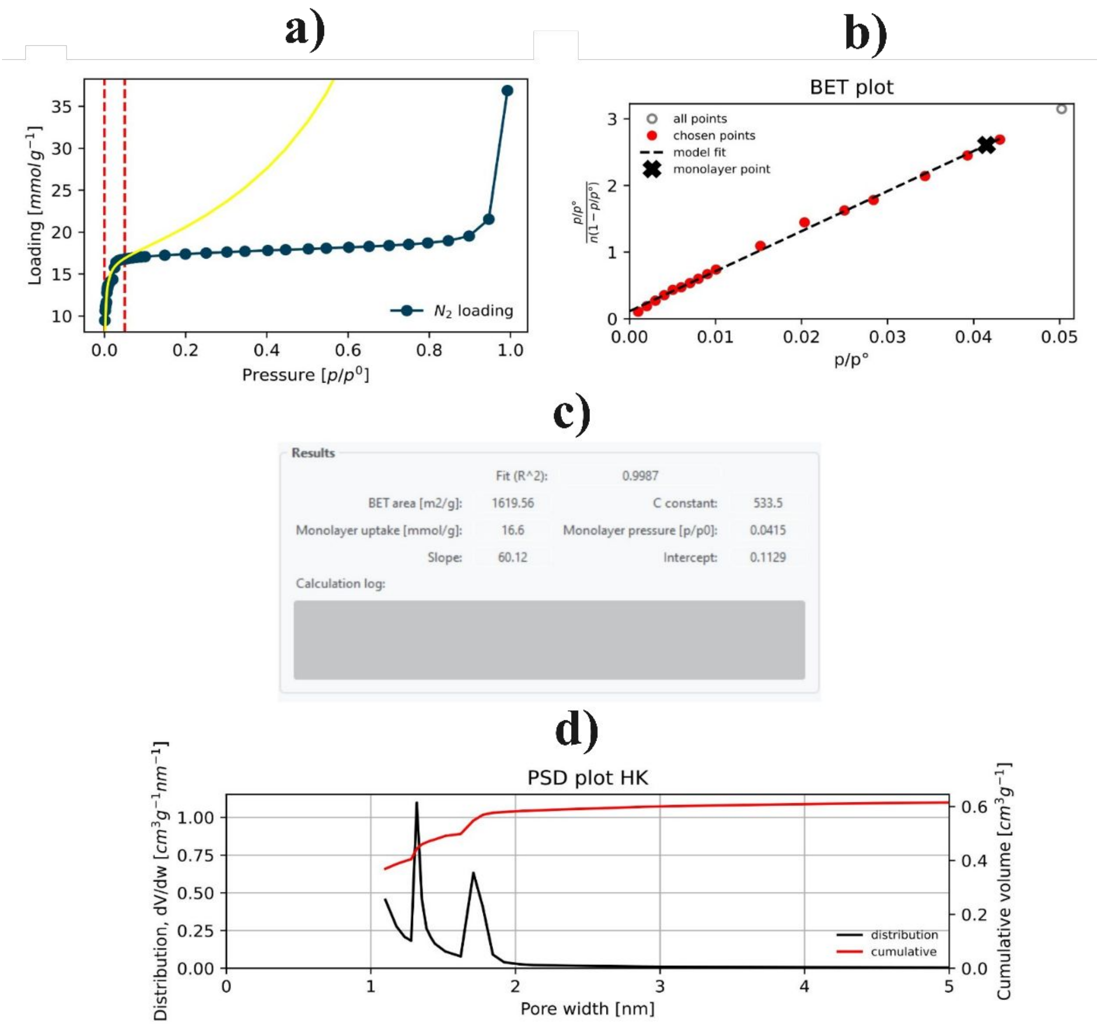

Figure S1: Physisorption results for ZIF-8 including a) isotherm b) BET plot c) BET model parameters and d) pore size distribution by HK method

	S_{BET}	Pore volume	Pore size
	(m²/g)	(cm^3/g)	(nm)
ZIF-8	1620	0.632	1.3

Table S1: BET surface area, pore volume, and pore size of ZIF-8 calculated using the HK method.

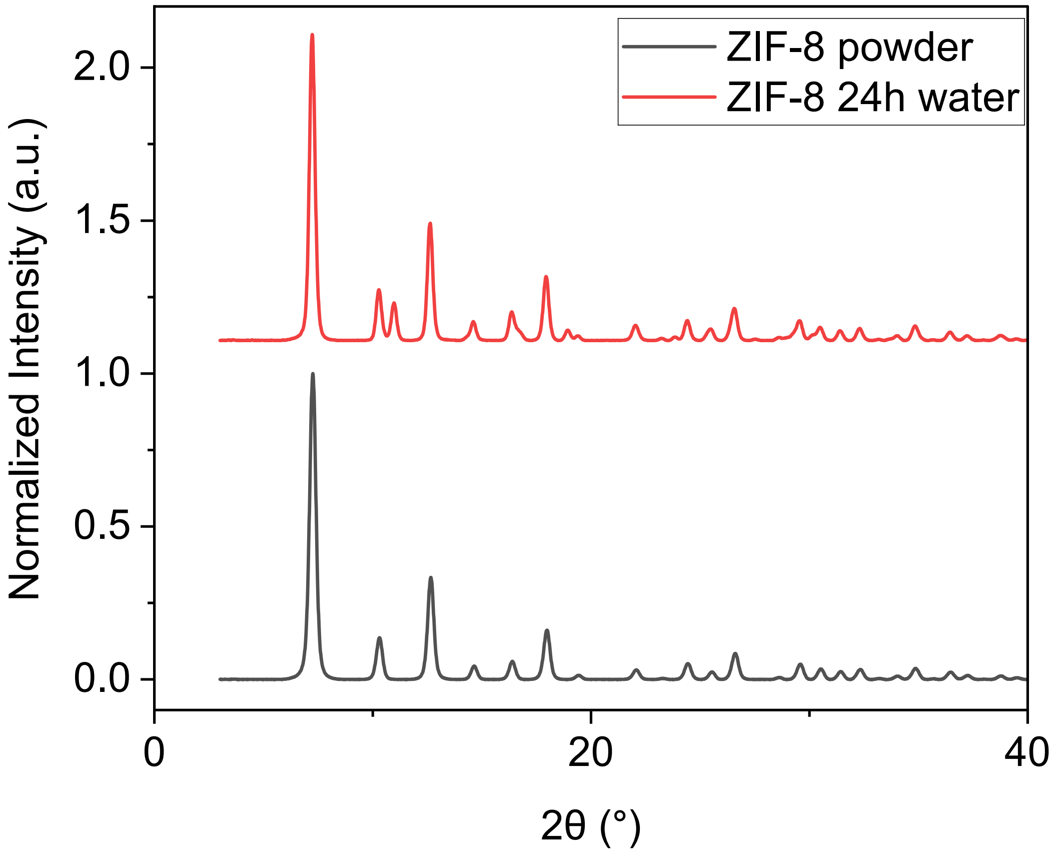

Figure S2: Diffractogram of ZIF-8 (powder) and ZIF-8 after 24h stirring in water at room temperature.

Material	Peak 1			Peak 2			Peak 3		
	x_c (2θ)	FWHM	Relative Intensity	x_c (2θ)	FWHM	Relative Intensity	x_c (2θ)	FWHM	Relative Intensity
ZIF-8	7.27	0.296	1	10.31	0.307	0.136	12.66	0.306	0.332
ZIF-8 water	7.43	0.272	1	10.29	0.286	0.164	12.63	0.281	0.394

Table S2: Results of peak fitting for ZIF-8 and ZIF-8 water exposition for 24h.

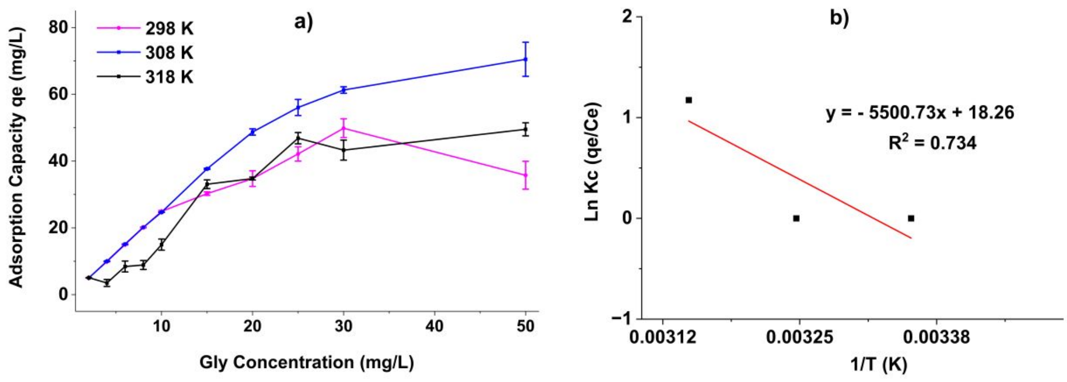

Figure S3. a) Effect of different temperatures in qe adsorption values; b) Van't Hoff plot of ln Kc (qe/Ce) vs 1/T (K) for glyphosate adsorption onto ZIF-8 for C_0 = 6 mg/L. Lines between points are for visual guidance only and do not indicate a mathematical trend. Kc is expressed in units L/g.
